# The impact of climatic factors on negative sentiments: An analysis of human expressions from X platform in Germany

**DOI:** 10.1016/j.isci.2025.111966

**Published:** 2025-02-07

**Authors:** Tareq Al-Ahdal, Sandra Barman, Stella Dafka, Barrak Alahmad, Till Bärnighausen, Michael Gertz, Joacim Rocklöv

**Affiliations:** 1 Heidelberg Institute of Global Health (HIGH), Heidelberg University Hospital, Heidelberg University, Heidelberg, Germany; 2 Interdisciplinar Center for Scientific Computing (IWR), Heidelberg University, Heidelberg, Germany; 3 Bioeconomy and Health, RISE Research Institutes of Sweden, Göteborg, Sweden; 4 Department of Environmental Health, Harvard T.H. Chan School of Public Health, Harvard University, Boston, MA, USA; 5 Department of Public Health and Clinical Medicine, Section of Sustainable Health, Umeå University, Umeå, Sweden; 6 Institute of Computer Science, Heidelberg University, Heidelberg, Germany

**Keywords:** Social sciences, Research methodology social sciences

## Abstract

Expressions in social media can provide a rapid insight into people’s reactions to events, such as periods of climatic stress. This study explored the link between climatic stressors and negative sentiment on the X platform in Germany to inform climate-related health policies and interventions. Natural language processing was used to standardize the text, and a comprehensive approach for sentiment analysis was utilized. We then conducted spatiotemporal modeling fitted using integrated nested laplace approximation (INLA). Our findings indicate that higher and lower level of temperature and precipitation is correlated with an increase and decrease in the relative risk of negative sentiments, respectively. The findings of this study illustrate that human sentiment of distress in social media varies with space and time about exposure to climate stressors. This emotional indicator of human exposure and responses to climate stress indicates potential physical and mental health impacts among the affected populations.

## Highlights

• Climate extreme events are linked to increased negative emotions from social media
• Temperature and precipitation levels correlate with the change in sentiments
• Social media can be used as an emotional indicator of climate stress

## Graphical abstract

**Figure:**
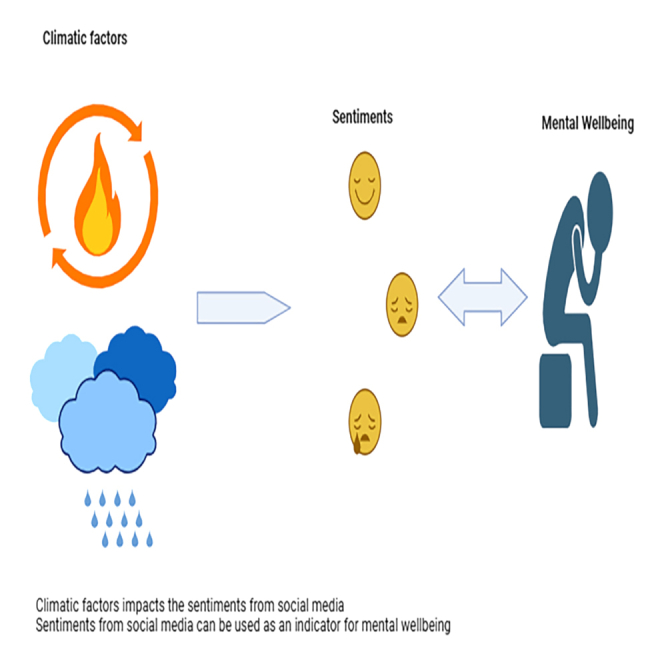

## Introduction

Emotional well-being is an important aspect of overall health^1^ and has been connected to various physical health consequences. Studies have shown that individuals with positive emotional states have stronger immune systems, decreased blood pressure, and a diminished likelihood of chronic disease progression, such as diabetes and heart disease.^2^^,^^3^^,^^4^ In contrast, negative emotional status has been associated with an elevated risk of compromised mental and physical well-being.^4^^,^^5^^,^^6^ Furthermore, the relationship between emotional well-being and other factors has been subject to much research in the previous years, though it is a complex and multifaceted issue.^7^

Social media sentences can be analyzed to understand the emotional orientation or attitude conveyed online, which can be classified as positive, negative, or neutral.^8^ Weather can give rise to stress through multiple pathways rainfall, for instance, can hurt our mood Research showed that individuals tend to feel less happy and more tired on rainy days compared to sunny days.^9^ This can be attributed, at least in part, to the association between gloomy weather and feelings of depression, isolation, and limited opportunities for social engagement and outdoor activities.^10^ In addition, extreme rainfall and floods can be dangerous and induce both physical^11^^,^^12^ and psychological stress.^13^

Other than precipitation, research has found that temperature can have an impact on expressed sentiment. People’s moods^14^ tend to be more positive on days with higher temperatures compared to lower temperatures. However, the relationship is not always linear and can be influenced by other factors such as humidity, wind, and season. Additionally, a study suggests that temperature increases above or below a certain comfort threshold tend to increase negative emotions such as anger, stress, and fatigue.^15^^,^^16^^,^^17^

Research has shown that less optimal weather conditions are associated with the deterioration of the conveyed sentiments.^18^ Baylis found a sharp decline in the hedonic state above 70°F,^19^ while temperature and hate speech online suggest that extreme temperatures either an increase or decrease can exacerbate the manifestations of aggressive tendencies online.^20^ Climatic content on the X platform was found to be significantly related to temperature in two hot cities,^21^ and previous indicators based on X platform data showed that local heat waves were associated with decreased positive and increased negative emotions.^22^ Extreme rainfall was also linked to increased negative expressions.^23^ In China, extreme weather events were associated with the worsening of the expressed perspectives online. Recent research from Spain found a strong negative effect of external temperature on the sentiments expressed on Twitter. This study underscores the relevance of our investigation that an increase in temperatures worsens the sentiments online.^24^ In addition, a study from China based on the analysis of sentiments and temperature, found that the positive sentiments increase at 15°C and 25°C.^25^ While most of the literature supports the idea that extreme temperature impacts individual sentiments, a recent study from China using geotagged posts from Weibo found that extreme low temperatures have a significant impact on social media sentiments but not extreme high temperatures.^26^

In contrast to existing research, our study applies a rigorous statistical spatiotemporal modeling frameworks using integrated nested laplace approximation (INLA) to analyze the association between climatic factors and negative sentiments.

In this research, we used X data from Germany by filtering the location and tweets with German language only to study the associations between negative sentiments and climatic factors, namely temperature, precipitation, and extreme flood events. To our knowledge, no other study has concentrated on the impact of climatic factors on negative sentiments within this geographic and time context.

## Results

The results presented here are derived from the sentiments analyzed using the LIWC-22 tool.

### Spatial analysis of precipitation, fatalities, and population distribution surrounding extreme flooding events

**Figure 1:**
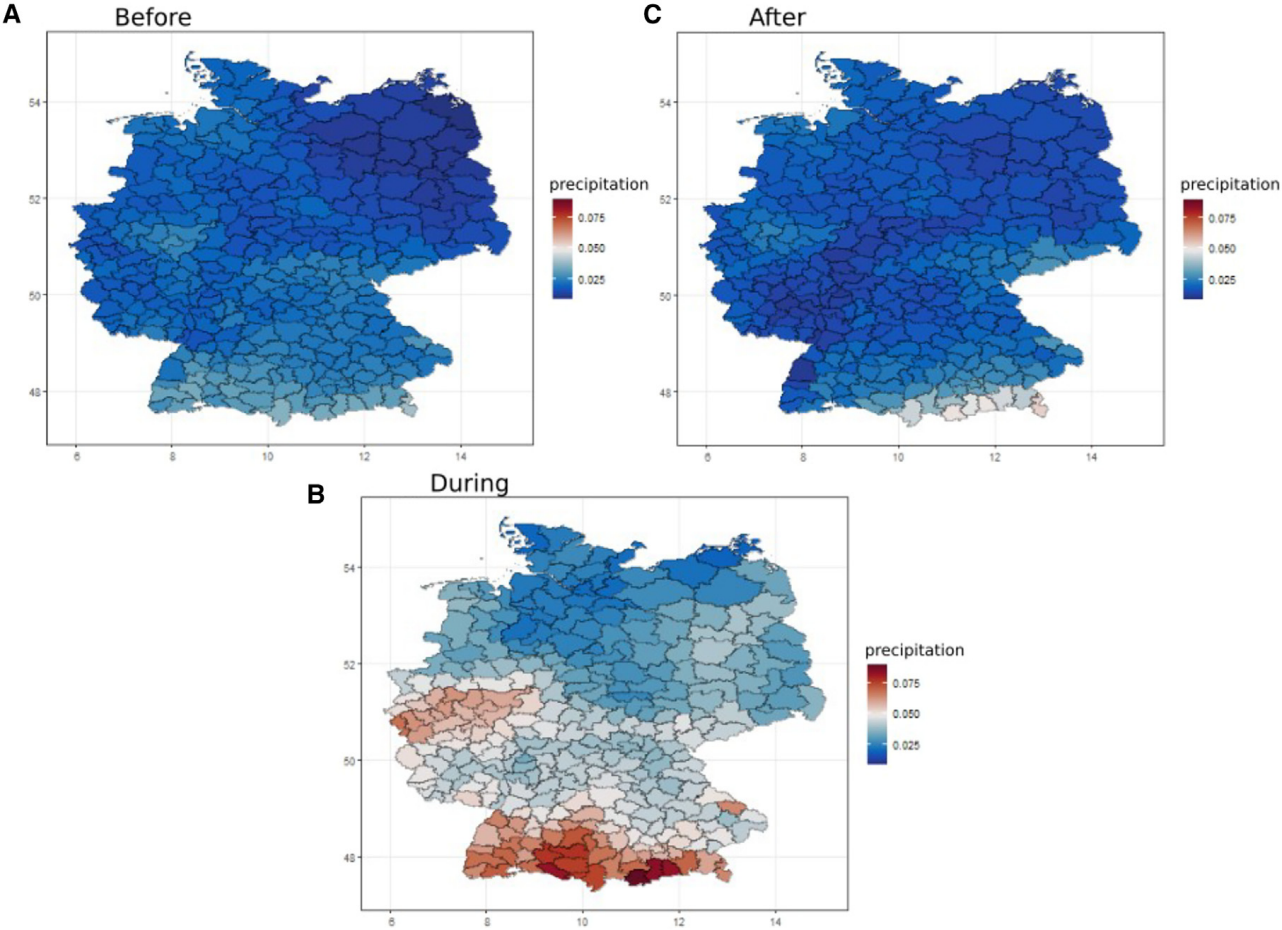
Spatial analysis of the precipitation (A–C) The total precipitation in (A) the weeks before the high precipitation event (2021-05-25–2021-06-21); (B) the weeks during the high precipitation event (2021-06-22–2021-07-19); and (C) the weeks after the high precipitation event (2021-07-20–2021-08-16). The color scale shows the magnitude of precipitation, with higher values depicted by red colors while lower values are represented by blue colors. The scale is the same in all three panels (A–C).

Precipitation patterns (Figure 1) highlight regions where the precipitation was prominent and high during the floods compared to before and after. It shows that the precipitation was high in the western and southern parts of Germany during the floods. High precipitation levels during the floods would have contributed to the flood events, as an excessive amount of precipitation in a short period causes runoff and leads to increased water levels in rivers and streams. The event period we identified in the four weeks between 2021-06-22 and 2021-07-19.

The mortality impact of the flood, portrayed in the map supplemental information (Figure S2), describes the distribution of fatalities, especially in the western region of Germany. Rhineland Pfalz stands as the most affected area with a staggering 134 deaths, followed by Nordrhein-Westfalen as the second most affected region with 48 deaths, and finally, Baden Württemberg, Bayern and Sachsen each reported a single death.

### Spatial statistical analysis of negative sentiments relative risk during extreme climatic event

**Figure 2:**
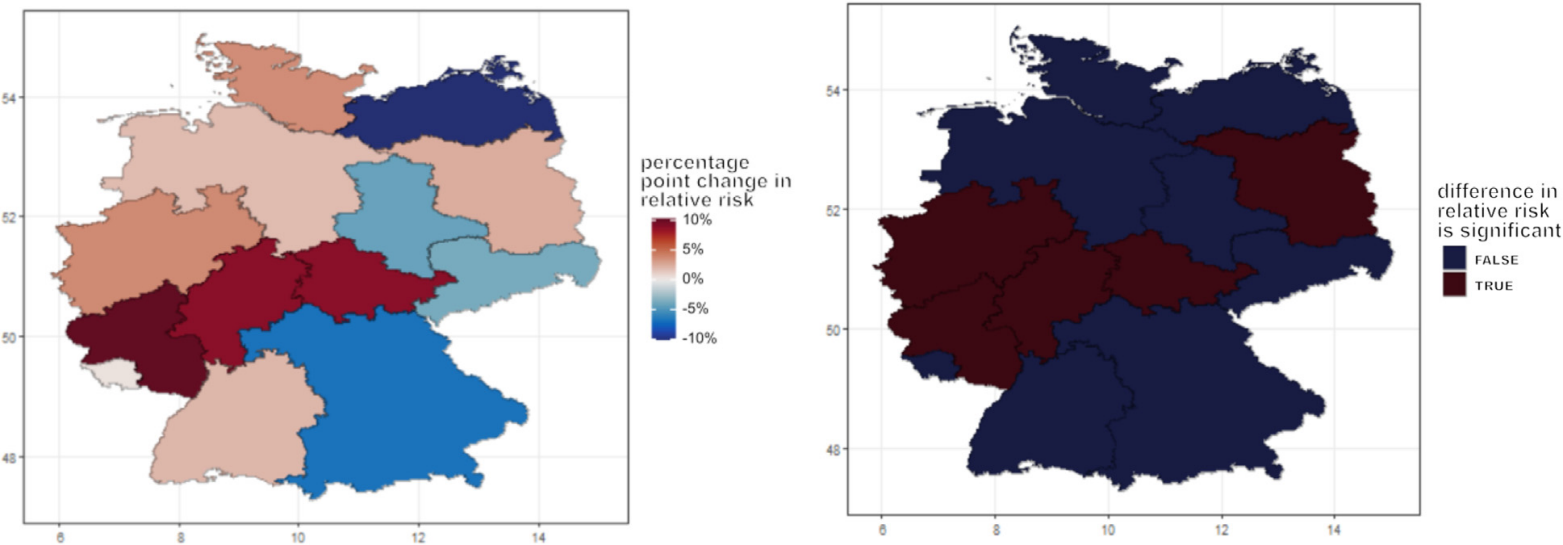
The spatial trend shows the difference in relative risk of negative sentiments before to during the high precipitation event The figure on the right side shows the difference in the increase in relative risk in the area exposed to floods is statistically significant. Further details can be found in supplementary materials S3 and S4.

We find a statistically significant increase in negative sentiment compared to baseline (Figure 2), in regions that were directly exposed to the flooding. The spatial trend underscores the far-reaching emotional impacts of flooding events with the negative emotions extending beyond the center and going beyond to surrounding regions during the event. The change in negative sentiment in each region is shown in supplemental information (Figures S3 and S4). This is the underlying data from which the change averaged over regions with/without fatalities.

The difference in relative risk of negative emotions from before to during the precipitation event, transitioning from the period of before (2021-05-25–2021-06-21) to during (2021-06-22–2021-07-19) reveals a statistically significant spatial effect. The difference in the relative risk increased up to 10%. The effect is prominent in the regions that bore the brunt of the flooding with a high number of causalities, the effect extends beyond the affected regions to the sounding areas. Statistical analysis confirms the significance of this spatial effect, underscoring the broader implications of the flooding event on the areas that are directly affected and neighboring regions.

Although we initially defined three distinct phases—before, during, and after the flooding for clarity when visualizing the amount of rainfall—our primary focus was on capturing the immediate emotional impact of the flooding event during the crisis comparing to patterns before and after.

### Impact of precipitation and temperature on negative sentiments

**Figure 3:**
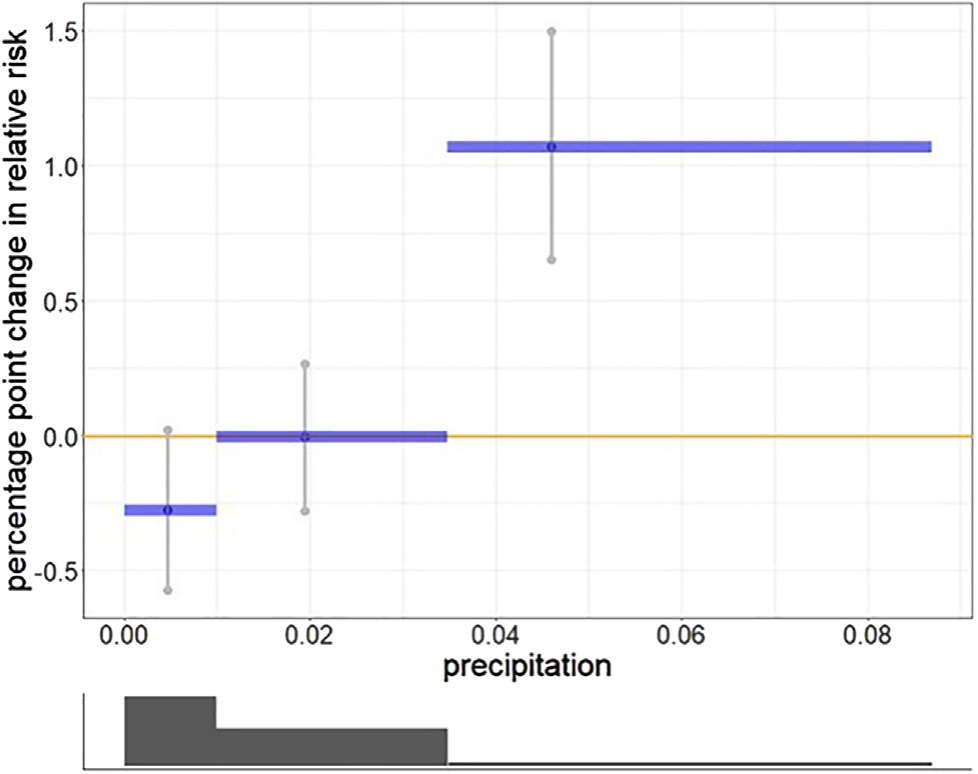
The non-linear random effect of weekly precipitation (blue graph) has been analyzed by categorizing the weekly precipitation in each region into three levels: low, mid, and high precipitation The precipitation categories are below 0.01; from 0.01 to 0.035; and 0.035 and higher (mm) (min = 0, max = 0.07). The light Gray graph represents the upper and lower bounds it shows that the effect is significantly below zero for the low precipitation level and significantly above zero for the high precipitation level. These upper and lower bounds are 95% significant bounds. The dark graph below shows the distribution of the sentiments in each category.

The illustration indicates that there is a significant difference in expressed negative sentiments between the low and high precipitation levels (Figure 3). Both the lower bound, and upper bound for low precipitation levels, and high precipitation levels is significantly a relative risk of 1, indicating that decreased precipitation levels are correlated with lower levels of expressed negative sentiments risk and, elevated precipitation levels are correlated with higher levels of expressed negative sentiments risk. The upper and lower bounds are 95% significance limits for the effect.

Overall, the findings suggest that there is a significant relationship between precipitation levels and expressed negative emotions, the increase of precipitation being associated with elevated levels of relative risk of negative sentiments up to 1.1% compared to the reference group, and the decrease of precipitation levels being associated with diminished levels of negative sentiments risk up to −0.25%.

**Figure 4:**
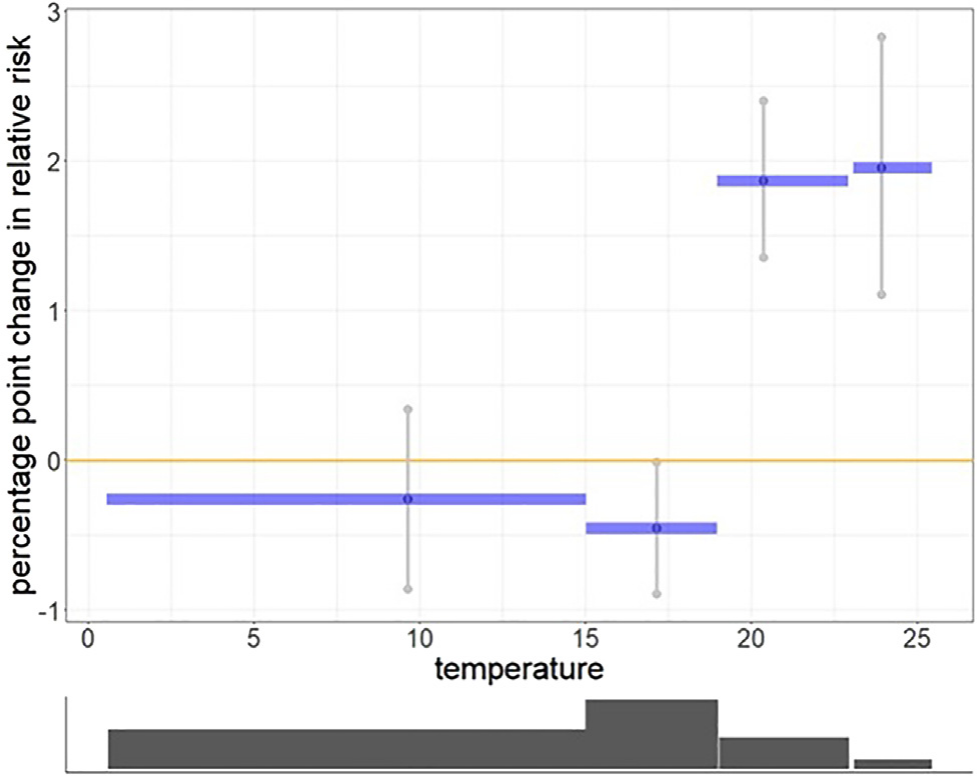
The non-linear effect of weekly temperature (blue graph) has been analyzed by categorizing the weekly temperature in each region into four levels: low, mid, average and high temperature The temperature categories are 0°C–15°C, 15°C–19°C, 19°C–23°C, 23°C and above, (min = −1, max = 24). The light Gray graph representing the upper and lower bounds it shows that the effect is significantly below zero for the low-temperature level second category and significantly above zero for the high-temperature level. These upper and lower bounds are 95% significant bounds. The dark graph below shows the distribution of the sentiments in each of the four categories.

The data visualization highlights a noteworthy distinction in the expression of negative sentiments concerning varying temperature levels (Figure 4). We observe that the lower bound for the low-temperature category falls significantly below zero, suggesting a decrease in the relative risk up to −0.5% in the second category indicating that lower temperatures are linked to a decrease in the expression of negative sentiments implying a protective effect rather than a harmful one. On the other hand, the upper bound for the high-temperature category extends significantly above zero, indicating that elevated temperatures above 20°C are linked with an increase in the expression of negative sentiments and the relative risk increased up to 2% in the fourth category. It’s important to note that these upper and lower bounds represent a 95% level of significance for the effect.

To sum it up, these results underscore a substantial relationship between temperature levels and the manifestation of negative emotions. Higher temperatures coincide with heightened negative sentiment risk, while lower temperatures are connected to a reduction in the risk of negative expressions.

## Discussion

This study provides a comprehensive exploration of the association between temperature, precipitation, flooding, and negative sentiment expressions. We utilized various processing techniques and natural language processing to perform sentiment analysis on more than 14.8 million tweets in Germany spanning the years 2019–2022. Our results suggest that higher levels of precipitation and temperature are associated with increased negative feelings. Conversely, lower levels of temperature and precipitation are associated with decreased negative feelings. Specifically, our study found that severe weather incidents, such as floods, exacerbate negative perspectives expressed on the X platform. The findings of this study have significant implications within the framework of climate change. Climate change has been correlated to adverse impacts on human outlook and behavior,^27^^,^^28^ increasing negative attitudes and actions that can exacerbate environmental and social problems. Tracking the emotional consequences of climate change on individuals and communities is crucial in developing effective solutions to alleviate and adapt to its effects.

The impact of temperature and precipitation on expressed negative opinions is a topic of growing interest in the field of climate change research.^29^ Our study supported other results from previous studies^18^^,^^19^^,^^22^^,^^23^ and has shown that fluctuations in temperature and precipitation can have significant effects on negative expressions. Additionally, extreme climatic events such as floods are associated with an increase in the expressed negative reactions which is also consistent with findings from other studies.^30^^,^^31^^,^^32^ Our analysis revealed that lower temperatures were associated with a decrease in negative sentiments, consistent with the relationship between emotional responses and climate factors. A study using geotagged posts from Weibo, one of China’s largest social media platforms, supports this finding, demonstrating that “extremely low temperatures causally decrease individuals’ sentiment”, this aligns with our results, suggesting that temperature fluctuations play a role in shaping negative public sentiment during heat stress.^26^ Our results are further supported by research conducted in Spain. A study analyzing Twitter data from 2017 to 2022 in Spain found a strong negative effect of external temperatures on sentiment, exacerbated by the increasing frequency of heat waves and climate-related deaths.^24^

The floods increased the expressed negative attitudes in the text data. Sentiment analysis of text data collected during and after a flood event can provide insights into the public’s perception of the event and its impact. In the case of a flood, people may express negative feelings such as fear, anxiety, frustration, or anger about the event and its aftermath. For example, people may express dissatisfaction with the response efforts of local authorities, concern for their safety and the safety of their loved ones, or frustration with the loss of property and belongings. They may also express anger about the causes of the flood, such as poor planning or lack of investment in flood mitigation measures. These negative views can provide valuable information for decision-makers and can inform future risk management and mitigation efforts. For example, understanding the sources of frustration and anger can help identify areas with possible mental risk and the need for early intervention.

The results in this research are consistent with prior studies that linked extreme temperature and precipitation to the increase in negative sentiments. All these findings from this research with previous findings at the experimental level collectively align with the previous assumptions that the variation in temperature and precipitation is associated with biological changes, such as the release of cortisol hormone,^33^^,^^34^^,^^35^ which is known to be linked to stress and mood regulation.^36^^,^^37^^,^^38^ In addition, temperature change can also trigger an inflammatory response^39^^,^^40^^,^^41^^,^^42^ in the body which is connected to an increased risk of depression and other mental health disorders. Other examples are an increase in temperature affects sleep and causes sleep disturbances^43^^,^^44^ and psychological stress^13^^,^^45^ Precipitation limits outdoor physical activity^46^ and physical activity is associated with improving mental well-being.^47^

This research also suggests scientists should conduct interventional studies that manipulate temperature and precipitation in controlled settings. Research with techniques that measure brain activity in response to the change in temperature and precipitation can provide insights into the neural mechanisms^48^ underlying the relationship between these factors and negative sentiments. In addition to traditional methods, utilizing advanced techniques such as virtual reality can offer a unique and immersive approach to measuring brain activity in response to temperature precipitation and extreme events.^49^

This study has several practical and policy implications. First, by examining how climate affects negative sentiments relative to risk, the study highlights the mental health consequences attributed to climate change, emphasizing the need for increased attention and resources for mental health services in communities that are affected by climate change. Moreover, it emphasizes the importance of investing in climate resilience and adaptation strategies that can help individuals address the health consequences of climate change. Additionally, highlights the significance of considering the emotional and psychological consequences of climate change, in addition to its physical effects. As such, addressing the emotional toll of temperature and precipitation fluctuations should be a fundamental aspect of any efforts aimed at mitigating and adapting to the impacts of a changing climate.

### Limitations of the study

Our analysis was based on a geotagged dataset from the X platform, which, while valuable for understanding spatial patterns of sentiment, may not be fully representative of the broader population. Geotagged tweets constitute only a small subset of all social media activity, and users who enable geotagging might differ demographically or behaviorally from those who do not. As a result, our findings might be biased toward the sentiments and behaviors of this specific group, which could limit the generalizability of our results. The German sentiment model which is trained on datasets that are representative of the target domain, sentiment analysis models are non-perfect, especially in cases where the context is complex. For example, sarcasm, irony, and figurative language can result in incorrect sentiments. It is also important to mention that machine learning algorithms are trained on fixed vocabulary that uses a predetermined collection of words or phrases to identify the sentiment of the text. As a result, it might miss important information if the text contains words or phrases that are not in the predefined vocabulary. Our analysis was limited to Germany, and we suggest that it is necessary to repeat the analysis in other countries to ensure generalizability.

Among the different limitations of the data, we mention here the outliers in social media data that might appear due to viral posts, controversial opinions, and abnormal behaviors so it is important to handle them properly to ensure the reliability of the results.

We can only establish correlations between the covariates, precipitation and temperature, and negative sentiments. However, correlation does not imply causation. It might be that precipitation or temperature is correlated with some other effects, such as time off from work, and that this effect is confounded with the covariates included in this work. We control missing variables in part with the spatial-temporal random field, but confounders could still have a sizable impact on the results. Further on, we acknowledge that the population on X may be a selection and may not be representative of the German populations’ emotional reaction to flood and weather events.

## Resource availability

### Lead contact

Requests for further information and resources should be directed and will be fulfilled by the lead contact, Joacim Rocklöv (joacim.rockloev@uni-heidelberg.de).

### Materials availability

This study did not generate new unique materials.

### Data and code availability

• Data: all data reported in this publication will be shared by the lead contact upon request.
• Code used in the analysis will be made publicly available immediately after publication through this link https://github.com/alahdalta1/Germany_Climate_Sentiment_Paper.git.
• Any additional information required to reanalyse the data reported in this article is available from the lead contact upon request.

## Acknowledgments

We acknowledge the support of the high-performance computing facility at the interdisciplinary center for scientific computing (IWR) for providing computational resources.

We acknowledge that The present contribution is supported by the 10.13039/501100009318Helmholtz Association under the joint research school “HIDSS4Health Helmholtz Information and Data Science School for Health”. Joacim Rocklöv, Tareq Al-Ahdal, and Sandra Barman received funding from the 10.13039/100005156Alexander von Humboldt Foundation.

## Author contributions

Conceptualization, T.A.-A., S.B., and J.R.; methodology, T.A.-A. and S.B.; software, T.A.-A. and S.B.; formal analysis, T.A.-A., and S.B.; investigation, T.A.-A.; resources, T.A.-A. and S.B.; data curation, B.A. and S.D.; writing—original draft, T.A.-A., S.B., and S.D.; writing—review and editing, T.A.-A., S.B., S.D., B.A., M.G., T.B., and J.R.; visualization, T.A.-A., S.B., and J.R.; supervision, M.G., T.B., and J.R.; project administration, T.A.-A; funding acquisition, J.R.

## Declaration of interests

The authors declare no competing interests.

## STAR★Methods

### Key resources table

**Table:** 

REAGENT or RESOURCE	SOURCE	IDENTIFIER
**Software and algorithms**		

LIWC 22	Pennebaker et al.^50^	https://www.liwc.app/
INLA	Lund et al.^51^	https://www.r-inla.org/
Python	(https://www.python.org/)	Python version 3.8.5
R	(https://www.r-project.org/).	*R version 4.0.3*
Codes used in the analysis	https://github.com/alahdalta1/Germany_Climate_Sentiment_Paper.git	Germany_Climate_Sentiment_Paper

### Method details

#### Data

We used the Centre for Geographical Analysis (CGA) dataset that contains tweets with geographical information such as GPS coordinates or location tags to be able to perform spatial analysis.^52^

#### Data processing

First, we crawled all the tweets from Germany by using the maximum and minimum latitude and longitude during the study period which is framed between the first of January 2019 and the first of August 2022. The total digital cohort used in this study after removing duplicates is 14,824,836 million tweets in Germany. Only tweets in the German language were included.

After the retrieval of tweets, the final sample undergoes natural language processing. In this preprocessing phase, we use natural languages toolkit libraries such as NLTK version 3.8.1, spaCY version 3.7.2, and TextBlob version 0.17.1 The regular expressions (Regex) version 2023.10.3 were used to eliminate punctuation, hashtags, user handles, multiple spaces, symbols, URLs, punctuations, HTML tags, non-alphabetical characters, and numbers. Then, NLTK was used for stop word removal, which involves removing common words, and tokenization, which is the division of the text into individual words. The preprocessing of the data was done by using Python. The data is then ready for sentiment analysis. In this step, we installed a German sentiment library,^53^ a machine-learning approach to perform sentiment analysis which is the process of determining the emotional tone in the texts. Further details are in the appendix and the method was previously published.

The same process was redone by using the LIWC22. It is a method that relies on a lexicon or dictionary of words and word stems associated with linguistic and psychological features. Further details are in the appendix and are published previously elsewhere.^54^ Initially, we used the German sentiment model for sentiment analysis, but it proved insufficiently accurate for our data. To improve accuracy, we tested our results with the LIWC22 sentiment model, which demonstrated high precision in reflecting sentiments. As a result, we adopted LIWC22 for our final analysis to ensure more reliable sentiment measurements.

We employed Shapely, a powerful library for geospatial analysis which enabled us to combine data based on their spatial relationships. The resulting attribute table contained data on negative, positive, and total sentiments for each polygon every week over a period of approximately 187 weeks spanning four years. The spatial analysis was performed on the federal state level, with the smaller regions Hamburg, Bremen, and Berlin joined with their neighbors.

#### Climatic data

The study used climatic data from the fifth-generation atmospheric reanalysis (ERA5) dataset to get information about precipitation and temperature from 2019 to 2022. This dataset is originating from the (ECMWF) which stands for the European Centre for Medium-Range Weather Forecasts and covers the period from 1959 onwards.^55^ The ERA5 dataset gives hourly estimates of many climate variables, such as temperature and precipitation, by combining data from models with measurements taken from satellites and instruments. The data covers the whole planet on a 30 km grid and reaches a height of 80 km. This information is accessible on the Climate Data Store provided by Copernicus Climate Change Service (C3S) at a resolution of 0.25° x 0.25°.^56^

We have categorized the flooding into three distinct time periods based on the calendar weeks of the year, covering the periods before and during the flooding. The first category, which spans between 25.05.2021 to 21.06.2021 corresponds to the time before the flooding. The second category covers the period during the flooding and includes days between 22.06.2021 to 19.07.2021. The third category covers the period after the flooding between 20.07.2021 to 16.08.2021.

We created three categories pre-flooding, during flooding, and post-flooding, primarily for visualizing precipitation trends over time. These categories help establish a clear timeline of the event. However, when analyzing the relative risk of the negative sentiment, our focus was on capturing the immediate emotional responses during the flooding itself. The pre-and post-flooding periods serve to frame the overall sentiment trend, but the core of the analysis revolves around the emotional impact experienced during the flooding.

#### Fatalities data

Records of fatalities related to the flooding were extracted from a study previously published^57^ The data of the fatalities were used as a visual insight to the realized health impact of the disaster.

#### Population data

The data used in the analysis was obtained from the Eurostat database. The "demo_r_pjanaggr3" dataset provides aggregated population statistics. The retrieval of this data was done by using R programming language.^58^

#### Statistical analysis (Modelling)

The general form of the model:

We used a Poisson generalized additive model (GAM) that aims to model the count of negative tweets in each region *i* at a given week *t*. The overarching model is formulated as follows:

NTw(i,t)∼Poisson(μ(i,t))

Where the mean function *μ* is a random effect modelled using non-linear functions of covariates precipitation and temperature. The Poisson distribution is a suitable choice for modelling count data, such as the number of tweets, where the count is a non-negative integer. In this model, the mean *(i,t)* represents the mean number of negative tweets in region i at week t. The Poisson distribution is based on the assumption that the count data is independent given the value of the mean function *μ*. Dependence within the data is modelled within the mean value function *μ*.

The mean (i,t) is modelled can be represented by the equation:

$$\mu \left(i,t\right)=\theta_{tot\;}\cdot TTW\left(i,t\right)\cdot relrisk\left(i,t\right)$$

The three components are:

1. $\theta_{tot\;}$, which is the ratio of negative to total number of tweets computed for the whole dataset. It represents the overall proportion of negative tweets in the dataset.
2. TTW(i,t), which is the total number of tweets in region i at week t. It represents the exposure or opportunity for negative tweets in region i at week t.
3. rel risk(i,t), which is the relative risk of negative tweets in region i at week t compared to the overall pattern represented by $\theta_{tot\;}\cdot TTW\left(i,t\right)$. The relative risk thus represents the departure from the overall pattern of negative tweets in region i at week t.

Writing the mean in the form $\mu \left(i,t\right)=\theta_{tot\;}\cdot TTW\left(i,t\right)\cdot relrisk\left(i,t\right)$ is a way to separate components of the expected number of negative tweets in region i at week t based on the overall proportion of negative tweets, the total number of tweets, and the relative risk of negative tweets in region i at week t compared to the overall pattern.

We use the standard link function of a Poisson GAM and model the logarithm of the relative risk as an additive random function of covariates:

$$\log\left(\text{rel}\;\text{riski},t\right))=spatio-temporal\;field\left(i,t\right)+\sum_{h}f_{h}\left({covariate}_{h}\left(i,t\right)\right)$$

The spatiotemporal field captures regional and seasonal variation in the risk, while the random effect $f_{h}$ allow for the effect of other factors, in our case weather conditions, to be incorporated into the model.

The spatio-temporal field can incorporate spatial and temporal variation that is not captured by the covariates. We model the spatio-temporal field using a Gaussian Markov random field with

1. an intrinsic conditional autoregressive (ICAR) or independent covariance structure of the random field in space, and
2. autoregressive process (AR) or independent process in time.

Using a spatio-temporal field with the ICAR covariance structure in space and/or AR process in time allows for dependence in space and/or time, such that values corresponding to regions that are close in the network (separated by only a few links) are more dependent than values corresponding to regions that are further apart. We use standard networks with a link between two nodes in the network if the corresponding regions are neighbours geographically, and links between consecutive time points.

The package R-INLA^53^ was used to fit the model. The ICAR is implemented in R-INLA as the Besag covariance structure.

Similarly, non-linear functions of the covariates (random effects) are defined using Gaussian Markov random vectors. Here, if the covariate indexed h can take n distinct values, denoted $x_{1},\ldots ,x_{n}$, we write the random effect $f_{h}=\left(f_{h}\left(x_{1}\right),\ldots ,f_{h}\left(x_{n}\right)\right)$, and define.

$$f_{h}∼N\left(0,C\right)\text{.}$$

The covariance C is defined by a chosen network, connecting nodes 1,…,n that represent the distinct values of the covariate. For a simple covariance, we define a network with links between consecutive values, and define either an AR process or an independent process on this network. When modelling interaction effects, we instead define a cross-covariate as the cross-product of two covariates, and a corresponding two-dimensional network with an ICAR covariance structure.

In particular, we classify precipitation into three groups: low, mid and high, with the random vector $f_{precip}=\left(f_{precip}\left(low\right),f_{precip}\left(mid\right),f_{precip}\left(high\right)\right)\text{.}$ For temperature, we classify temperature into four groups: low, mid, average and high.

### Quantification and statistical analysis

#### Software and approach

The INLA (Integrated Nested Laplace Approximation) method was implemented using R version 4.0.3 (R Foundation for Statistical Computing). The INLA package (version 20.01) was used to run the Bayesian model and perform statistical inference. The model was run on the IWR high-performance computing servers.

Python: Data cleaning and preprocessing of the textual data were carried out using Python version 3.8.5. Various Python libraries (e.g., pandas, numpy, and regex) were used to preprocess and organize the textual data before analysis.

Computational Resources: The data was processed and analyzed using IWR high-performance computing servers, which provided the necessary computational power for running the model and handling the large datasets involved in this study.

#### Statistical method

We used a Poisson generalized additive model (GAM) to model the relation between negative sentiments and climatic factors.

#### Imaging

All figures in the paper were created using RStudio, with resolution adjustments made in Adobe Photoshop. The Graphical Abstract was designed using the free version of BioRender.
