# Supplementary material for: The impact of climatic factors on negative sentiments: An analysis of human expressions from X platform in Germany
Source: iScience. 2025 Feb 7;28(3):111966. doi: 10.1016/j.isci.2025.111966 (PMC11926722; doi:10.1016/j.isci.2025.111966)
Supplement: Document S1. Figures S1–S6 and Data S1 [file mmc1.pdf]

iScience, Volume *28*

Supplemental information

The impact of climatic factors on negative sentiments: An analysis of human expressions from X platform in Germany

Tareq Al-Ahdal, Sandra Barman, Stella Dafka, Barrak Alahmad, Till Bärnighausen, Michael Gertz, and Joacim Rocklöv

This document provides **Supplementary Material Figure Legends with Data S1**

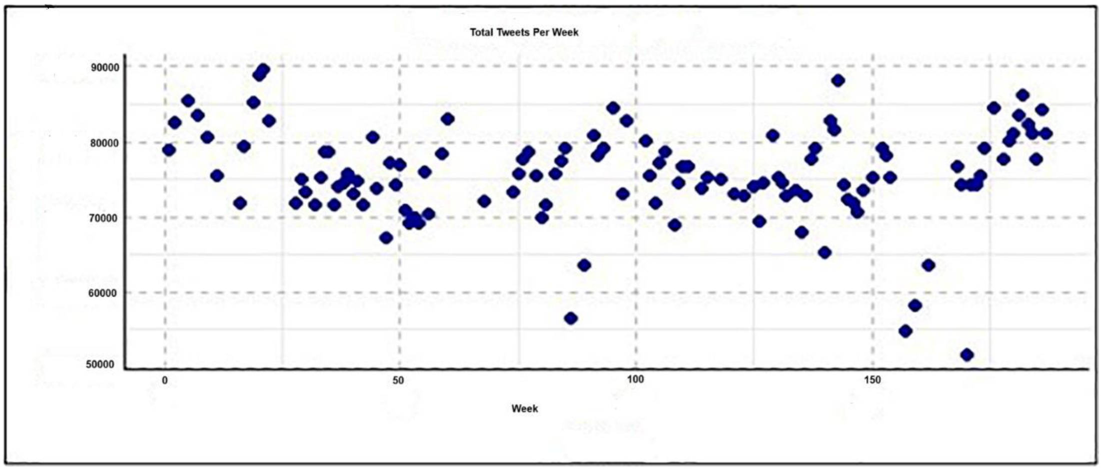

Figure S1: A temporal analysis of tweets activity over the weeks

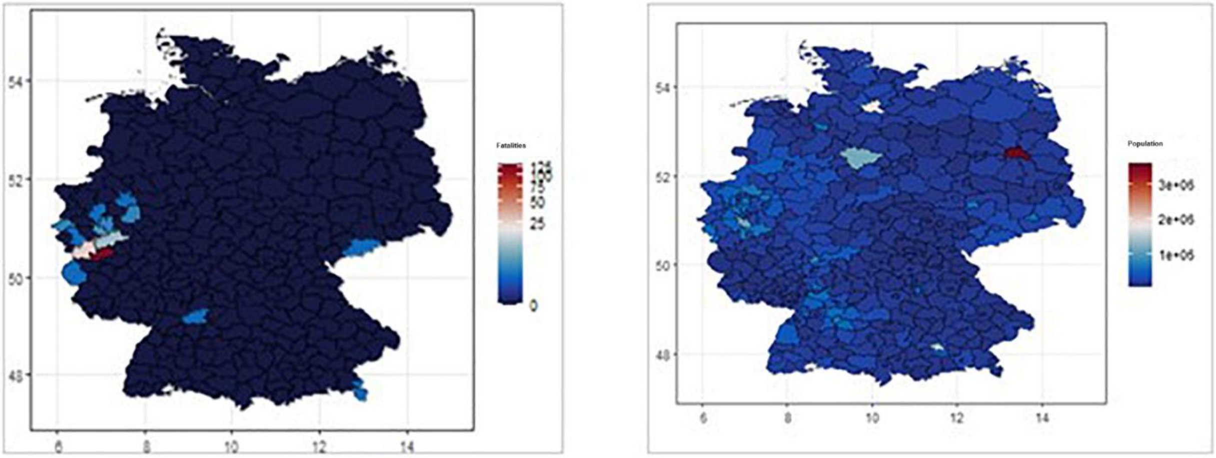

Figure S2: Fatalities in the flooding after the high precipitation event (left) alongside the population map (right). Note that the color scale is nonlinear in the fatalities map.

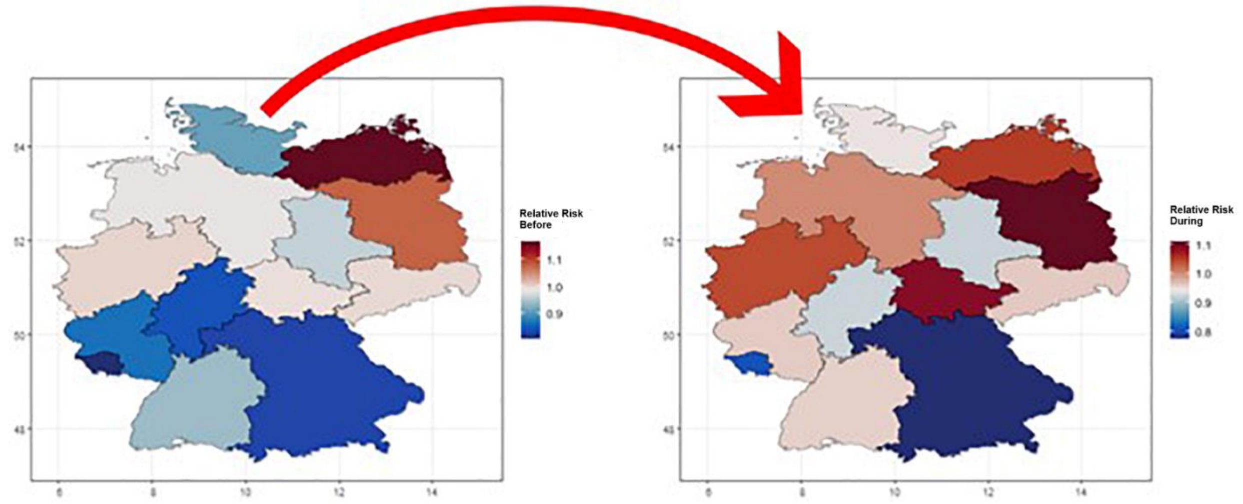

Figure S3: Relative Risk of Negative Tweets Before and During the Flooding Event. Highlighting significant increases across all regions, with Rheinland Pfalz bearing the highest impact

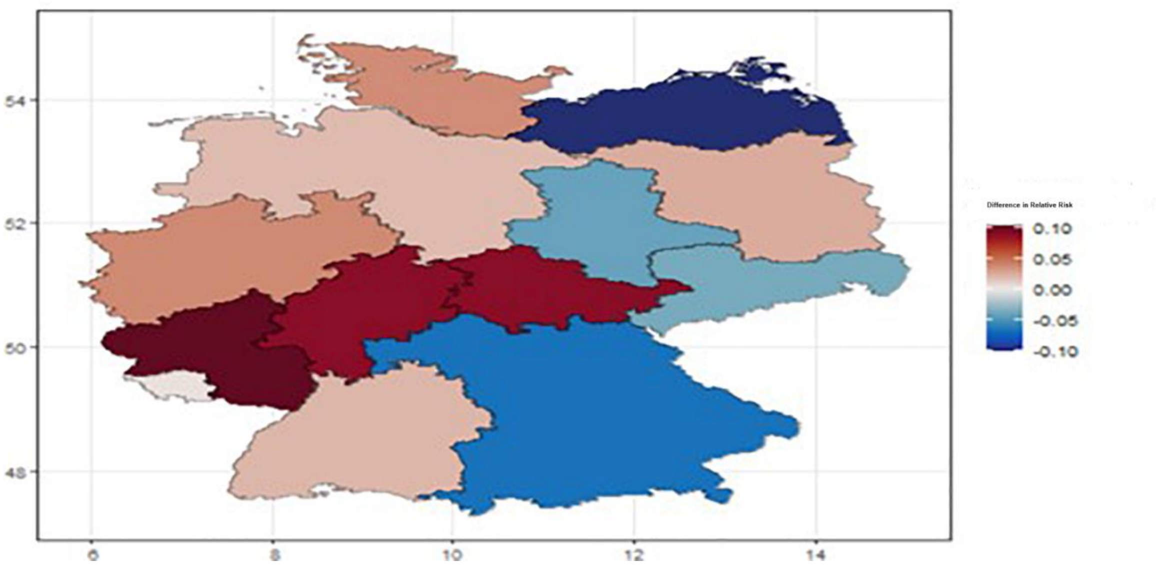

Figure S4: Relative risk before during and after the high precipitation event.

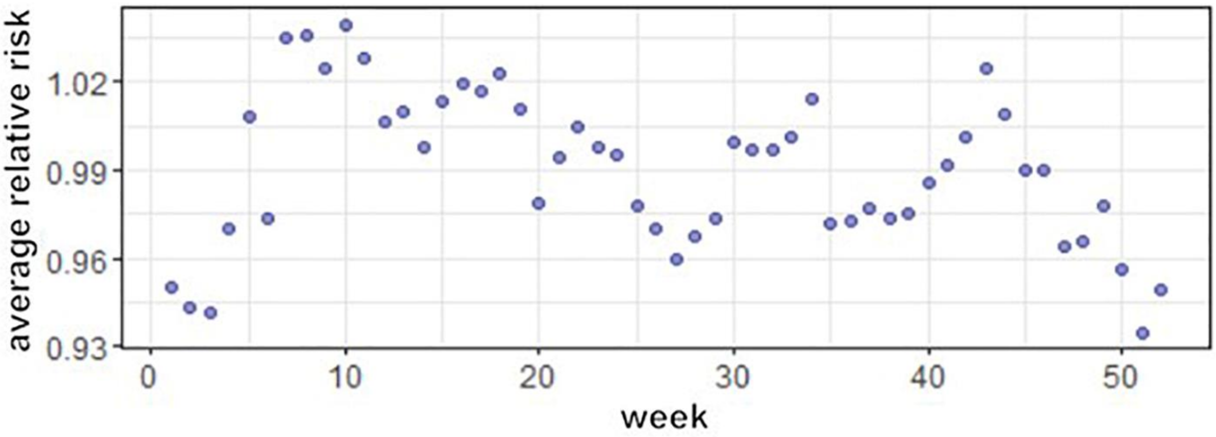

Figure S5: A temporal analysis of average relative risk showing the peak during the year 2020.

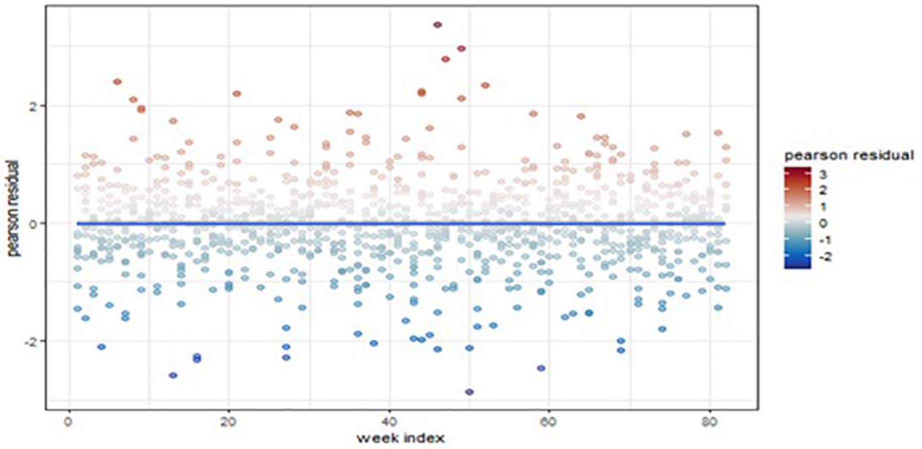

Figure S6: A temporal analysis of averaged Pearson residuals over time.

Data S1

Sentiment analysis approaches

a. The machine learning approach “the German Sentiment Model”

This machine-learning model was trained on 1.834 million samples using text from Twitter, Facebook, movie, or hotel reviews. The sentiments of this analysis are classified as positive, neutral, or negative. We used high-performance computing to process the dataset. Tweets were aggregated weekly using an attribute table. [1]

b. The dictionary-based approach” Linguistic inquiry and word count”

The construction of this tool was based on a multi-step process from the collection of words and judge rating phases to base rate analyses and psychometric evaluations. It is worth noting that this tool was validated on 15000 text samples to ensure this tool remains reliable and scientifically sound for linguistic analysis Further details are published previously in. [2]

Data Privacy

Several measures were taken to address data privacy concerns. First, we ensured that all personal identifiers were removed from the dataset. Second, the analysis was conducted on aggregated data rather than individually. Also, we implemented secure data handling practices with restricted access to the data. Finally, we will not make the full dataset publicly available, we will provide the aggregated and anonymized data that supports the findings of this study.

Climatic Data

The hourly accumulated precipitation fields were summed to create 24-hour precipitation totals, while the temperature was calculated as a daily mean. Subsequently, weekly mean values were computed from the daily values for all ERA5 fields at each station in Germany spanning the period of 2019-2022. The nearest neighbour approach was employed to identify the closest ERA5 grid point at the station level. Despite its usefulness, reanalysis poses significant challenges in reconstructing climate variables, such as precipitation, which exhibit large variations in both time and space.[25] Previous studies have identified enhancements in the representation of global-mean rainfall in the latest version of the European Centre for Medium-Range Weather Forecasts (ECMWF) atmospheric reanalysis dataset (ERA5) relative to its predecessor, ERA-Interim. These improvements were demonstrated by comparing ERA5 with the Global Precipitation Climatology Project (GPCP). Furthermore, a more recent investigation evaluated the performance of ERA5 precipitation globally by comparing it to observations and found that ERA5 spatial precipitation patterns demonstrated good agreement with observations, particularly for the European flooding event in 2021. These results suggest that ERA5 can accurately capture extreme rainfall events over the extratropic. [3]

Temporal analysis

The overall spatial trend shows that there is a steady increase and then decrease in the total number of tweets within a range of 70000 to 85000 tweets **(Figure S1)**. However, the figure also captures some outliers either by increase or decrease around certain weeks. We also went back to the original data and checked these certain anomalies which we assumed could be due to certain factors for example tweet activity increases during specific occasions, celebrity engagement,

breaking news, and promotional campaigns. The tweet activity also drops due to technical issues on weekends and holidays, and a lack of trending topics.

Shapefile and Spatial aggregation

For the analysis conducted on the Germany-specific data, spatial aggregation was performed at three different administrative levels: NUTS3, NUTS2, and NUTS1. Utilizing a shapefile containing the geographical boundaries of these NUTS regions, we accurately associated sentiment data with the appropriate administrative units. The shapefile used was sourced from the official NUTS boundaries provided by Eurostat, ensuring consistency with regional definitions. This hierarchical aggregation allowed for a detailed examination of sentiment variations across Germany, from the local (NUTS3) to broader regional (NUTS2) and national levels (NUTS1)

Indirect Standardization of X data

- To standardize the negative sentiment tweets between different locations (X data), we used the following formulas
- $e_{(i,t)} = p_{(i,t)} * R$
- $R = \sum y_{(i,t)} / \sum p_{(i,t)}$

Where:

- $e_{(i,t)}$: Expected sentiments for a county *i* at time *t*
- $p_{(i,t)}$: Population of the county i at time *t*
- R: Rate from the reference population (e.g., statewide).
- $y_{(i,t)}$: Number of sentiments in county *i* at time *t*

Data Normalization

The INLA spatiotemporal model is flexible in that it can take input data of different kinds, such as continuous data (climatic data) and categorical data (sentiment categories), and create a joint

model framework. We discretized the climatic data to obtain the climate covariates used in the paper. A strength of this modelling framework is that we can fit non-linear random effects, making the model relatively independent of the scale and normalization of the covariates. Normalization still matters in how we create the categories for the covariates, but less so than if we were to use e.g. a linear or quadratic model. It is also important how we aggregate the X data, in that it is good to have a relatively equal number of tweets in each region. The number of tweets is considered in the model, but model performance is improved if the regions have a similar number of tweets. We get more stable model performance when working on the NUTS2-level as compared to the NUTS3-level, due to the larger number of tweets within each region.

Complexity Analysis

The complexity of our analysis stems from multiple computationally intensive steps. Initially, we performed Natural Language Processing (NLP) on large datasets using Python libraries such as NLTK and Regex for data cleaning, tokenization, and text processing. Following this, sentiment analysis was conducted using the LIWC22 tool to extract sentiment indicators from the processed text. These steps, combined with the size of the dataset, involved significant computational resources.

Subsequently, the final sentiment data, along with climatic and spatial-temporal variables, was modeled using Integrated Nested Laplace Approximation (INLA). The application of Integrated Nested Laplace Approximation (INLA) in our spatiotemporal framework involves significant computational resources due to the complex nature of Bayesian inference and the large dataset. However, the INLA framework is efficient in that it uses sparsity in the model to make computations efficient.

The relative risk of the negative tweets from before to during the flooding event

The spatial dynamics of the study location before and during the flooding event **(Figure S3)** according to the definition see the Methods section. There was a significant increase in the relative risk in all regions during the flooding event, specifically in the Rhineland Pfalz which was the most affected by floods with the highest number of causalities.

The difference in relative risk before during and after the high precipitation event

The illustration shows the distribution of relative risk among all regions and the highest relative risk was in the area of flooding and its neighboring regions **(Figure S4)**.

2020 was excluded due to COVID-19 restrictions.

We decided to exclude the year 2020 from the analysis **(Figure S5)**. The rationale behind this decision is that due to COVID-19 restrictions have a significant impact on emotions. This significant increase in the positive and negative emotions made it an outlier in the analysis. In order to ensure the integrity and accuracy of the results, it was essential to remove them this year. By removing the data from 2020, the analysis could provide a more meaningful understanding throughout the rest of the years under consideration.

Model accuracy using Pearson Residuals

As part of the model validation process, we used **Pearson residuals** to assess the model fit. **Figure S6** shows the Pearson residuals plotted over time for each data point. Most data points fall within

the range of +3 and -3, indicating that the model generally fits the data well. However, there are a few points where the residuals exceed +3, suggesting potential outliers or areas where the model's predictions deviate more significantly from the observed values.

Despite these outliers, no systematic bias or temporal patterns are evident in the residuals, supporting the overall robustness of the model across time. The residuals indicate that the model performs well for most of the data points, with only minor deviations.

Unexplained variation

The variation due to random effects (unexplained by the covariates) is relatively large when compared to the variation explained by the covariates suggesting that covariates do not fully account for the significant amount of unexplained variability in our data. Similarly, the variation between regions is large compared to the variation explained by the covariates. This finding is important to consider in model building and in the interpretation of the results.

Future Recommendations

In this work, we emphasized the importance of future work to combine interdisciplinary approaches from different fields such as psychology, computational social science, and environmental science, public health to gain a comprehensive understanding of the issue. Longitudinal studies are also required to track changes in public sentiments over time. Extreme weather changes highlight the need for the development of indicators of negative sentiments related to health, and climate nexus. Further research should consider marginalized communities

and individuals who might be impacted by the effects of climate change. Finally, solutions will require collaboration between academic researchers, industry leaders, and policymakers to translate research findings into concrete actions and strategies to mitigate the negative impacts of climate change.

Supplementary References

1. Guhr, O., Schumann, A.-K., Bahrmann, F., and Böhme, H.J. (2020). Training a broad-coverage German sentiment classification model for dialog systems. pp. 1627-1632.
2. Boyd, R.L., Ashokkumar, A., Seraj, S., and Pennebaker, J.W. (2022). The development and psychometric properties of LIWC-22. Austin, TX: University of Texas at Austin, 1-47.
3. Hersbach, H., Bell, B., Berrisford, P., Hirahara, S., Horányi, A., Muñoz-Sabater, J., Nicolas, J., Peubey, C., Radu, R., and Schepers, D. (2020). The ERA5 global reanalysis. Quarterly Journal of the Royal Meteorological Society *146*, 1999-2049.
